# Towards sustainable local welfare systems: The effects of functional heterogeneity and team autonomy on team processes in Dutch neighbourhood teams

**DOI:** 10.1111/hsc.12604

**Published:** 2018-07-26

**Authors:** Alissa Lysanne van Zijl, Brenda Vermeeren, Ferry Koster, Bram Steijn

**Affiliations:** ^1^ Erasmus University Rotterdam Rotterdam Netherlands

**Keywords:** boundary management, functional heterogeneity, information elaboration, neighbourhood teams, team autonomy, team cohesion

## Abstract

Nowadays, many European countries delegate health and social care responsibilities from the national level to local authorities. In January 2015, the Netherlands similarly introduced a policy programme authorising municipalities to set their own social welfare policy. A specific feature of this programme is that it stimulates municipalities to implement teams wherein professionals from different disciplines are collectively responsible for a team's decision‐making. This suggests that teams ideally have (a) high levels of *functional heterogeneity* (professionals from different disciplines) and (b) high levels *of team autonomy* (collective responsibility and decision‐making). Based on the policy programme, it can be further assumed that (a) *information elaboration*, (b) *boundary management* and (c) *team cohesion* in teams will improve. In practice, the majority (87%) of Dutch municipalities implemented neighbourhood teams in January 2015. A common feature of these neighbourhood teams is that the various professionals are collectively responsible for all the curative and preventive healthcare, social work and voluntary social support of the citizens in a specific neighbourhood. Nevertheless, the structure and organisation of neighbourhood teams (including the level of functional heterogeneity and team autonomy) vary within and between municipalities. Given this situation, our aim was to examine to what extent functional heterogeneity and team autonomy influence information elaboration, boundary management and team cohesion in neighbourhood teams. We developed six hypotheses based on literature that were then tested on data collected (between May 2016 and January 2017) through an online survey from 1335 professionals in 170 neighbourhood teams. An SEM analysis showed a positive effect of team autonomy on information elaboration, boundary management and team cohesion. Results further showed a negative effect of functional heterogeneity on information elaboration and boundary management. The implications of these findings for practitioners and academics are discussed.

**What is known about this topic**

- Scholars in social and healthcare literature have suggested that cross‐professional collaboration in teams leads to sustainable care.
- Additionally, sustainable care is assumed to improve when team members manage their resources collectively to meet the specific needs of the population they serve.
- Information elaboration, boundary management and team cohesion are characterising high‐performing teams in the public sector.

**What this paper adds**

- Adding to the team literature, we offer theoretical foundations for propositions on how team autonomy can affect team outcomes through information elaboration, boundary management and team cohesion.
- Adding to the current debate on team heterogeneity, we find a negative relationship between functional heterogeneity and information elaboration.
- Team autonomy seems to be a powerful intervention in policy programmes that aim to improve team working.

## 1 INTRODUCTION

Prior to January 2015, social and healthcare professionals in the Netherlands were typically employed in fragmented, sector‐ and discipline‐oriented, regional organisations (Dijkhoff, 2014). As fragmentation is often associated with inefficiency, the Dutch government implemented a policy programme entitled “welfare reform for sustained care in the social domain” that decentralises social and healthcare responsibilities to local governments, combined with a strong focus on integrated working (Dijkhoff, 2014; SCP, 2015). The general objective of this programme is “one family ‐ one plan ‐ one director”, stimulating professionals from various disciplines to bundle their expertise in a single coherent approach (SCP, 2015). This implies that professionals from different disciples come to shared decisions and diffuse responsibilities. With this in mind, the programme stimulated municipalities to implement multidisciplinary teams (SCP, 2015; Van Rijn, 2013, 2014). Two important features of these teams would then be (a) *functional heterogeneity* (e.g. professionals from different disciplines) (Jackson, 1992) and (b) *team autonomy* (collective responsibility and decision‐making) (Uhl‐Bien & Grean, 1998).

Underlying the policy programme, we furthermore identify three assumptions about professionals’ cooperative behaviours (Dijkhoff, 2014). The first assumption is that professionals will become more familiar with the citizens’ needs (Dijkhoff, 2014). In order to become familiar with the citizens’ needs, the professionals are expected to take part in a process that involves: (a) exchanging information and perspectives with other professionals within the team, (b) individually processing the information and perspectives, (c) feeding back the results of this processing to the other professionals in the team and (d) discussing and integrating the final implications. In the literature, this process is often referred to as information elaboration (Van Knippenberg, Dreu, Carsten, & Homan, 2004). The second assumption is that professionals will optimise their communication and cooperation with relevant stakeholders (Dijkhoff, 2014). This implies that the professionals will manage the relationships with external stakeholders (i.e. organisations, clients, advisors and government) who provide information to, or absorb information from, the team. This is also known as boundary management (Gladstein, 1984). The third assumption is that professionals will create coherent local policies that enable them to work efficiently and interdependently. In developing coherent local policies, the professionals are expected to become and remain united to achieve their shared instrumental objectives, which is also defined as team cohesion (Tekleab, Quigley, & Tesluk, 2009).

As soon as the policy programme was implemented, the majority (87%) of Dutch municipalities employed professionals in *neighbourhood teams* (Movisie, 2016). These neighbourhood teams commonly consisted of a range of professionals (e.g. social worker, community psychiatric nurse, psychologist, youth worker) collectively responsible for the social work and curative and preventive healthcare of citizens in a specific neighbourhood (Dijkhoff, 2014; Thylefors, Persson, & Hellström, 2005). The structure and organisation of these neighbourhood teams varied across and within municipalities. They have different levels of functional heterogeneity and team autonomy. Consequently, we can examine to what degree functional heterogeneity and team autonomy influence information elaboration, boundary management and team cohesion. The main research question of this article is thus:

To what extent do functional heterogeneity and team autonomy influence informational elaboration, boundary management and team cohesion in Dutch neighbourhood teams?

Through answering this question, this article will provide two main contributions to the literature and one practical contribution. The first contribution is to the social and healthcare literature. By integrating team literature with the social and healthcare literature, the present article contributes to a better understanding of the role of team complexity in the social and healthcare context. The second contribution is to the team literature where this study particularly responds to calls to examine the relationship between a team's characteristics and its subsequent processes and emergent states, rather than between a team's characteristics and its outcomes (Mathieu, Maynard, Rapp, & Gilson, 2008). Here, the present study offers theoretical foundations for future hypotheses on how functional heterogeneity and team autonomy influence team outcomes through information elaboration, boundary management and team cohesion. Finally, the present study also makes a practical contribution. By examining the assumptions underlying the policy programme, this article provides a theoretical underpinning to the policy programme that will help policymakers optimise the operationalisation of the programme (Bickman, 1987).

The structure of this article is as follows. We start by discussing theory and develop six hypotheses. Next, we discuss the methods used to test these hypotheses and present the results of our analyses. Finally, we elaborate on the implications in the discussion section.

## 2 THEORETICAL FRAMEWORK

### 2.1 Neighbourhood teams

For several decades, teams have been implemented in a broad range of human service organisations (Kennedy, Armstrong, Woodward, & Cullen, 2015; Øvretveit, 1997). Teams can be viewed as “a collection of individuals who are interdependent in their tasks, share responsibility for outcomes, see themselves and are seen by others as an intact social entity embedded in one or more larger social systems, and manage their relationship across organisational boundaries” (Cohen & Bailey, 1997, p. 241). Despite sharing these features, there are many different types of teams (Cohen & Bailey, 1997; Katzenbach & Smith, 1993) of which the “neighbourhood team” is the specific variant studied in this article.

In studying teams, researchers commonly rely on modified versions of the “input – process – output framework” (I‐P‐O) that was introduced by McGrath (1984). This framework argues that a team's input influences the outputs through team processes. Despite its widespread application, the I‐P‐O framework has been criticised for oversimplifying team complexity and, accordingly, it has been recommended that researchers should predominantly focus on the effect of a team's inputs on the subsequent processes and emergent states that mediate the effect of these inputs on team outcomes (Ilgen, Hollenbeck, Johnson, & Jundt, 2005; Mathieu et al., 2008). Taking this into account, the present study focuses on the relationship between the *inputs* and the *processes and emergent states* of neighbourhood teams to gain initial insights into their complexity.

### 2.2 Information elaboration, boundary management and team cohesion

Information elaboration, boundary management and team cohesion are characterising high‐performing teams in the public sector (Kuipers & Groeneveld, 2014). More specifically, information elaboration covers the process of (a) exchanging information and knowledge, (b) discussing the various perspectives and (c) integrating the information and perspectives (Van Dick, Van Knippenberg, Hägele, Guillaume, & Brodbeck, 2008). Then, the boundary management process represents the team members’ active management of the team's relationships with external stakeholders (Dijkhoff, 2014). Boundary management relates to information elaboration, building on the idea that teams match their information process capacity to the information‐processing that their stakeholders request (Ancona & Caldwell, 1992a). Finally, team cohesion grasps the tendency for a team to develop and maintain high levels of unitedness towards the team's instrumental objectives (Tekleab et al., 2009). In line with the idea that information elaboration, boundary management and cohesion are beneficial for team outcomes in a public sector context, also the Dutch policy programme assumes beneficial effects for team outcomes in the health and social care context (Dijkhoff, 2014). Important to note is that cohesion is conceptually different from information elaboration and boundary management since cohesion is an *emergent state* while the latter two are *processes* of the team (Marks, Mathieu, & Zaccaro, 2001). Consequently, the two team *inputs* that the policy programme emphasises are functional heterogeneity and team autonomy (Dijkhoff, 2014). These are discussed in more detail below.

### 2.3 Functional heterogeneity

A basic hypothesis in the social and healthcare literature is that cross‐professional collaboration is essential for sustainable care, and will be better organised within a single team rather than across different teams (Jones, Bhanbhro, Grant, & Hood, 2013; Thylefors et al., 2005). Various organisational roles are represented in these cross‐professional teams, meaning that the team is functionally heterogeneous (Jackson, 1992). The actual level of functional heterogeneity in a team depends on the number of different job roles relative to team size (Keller, 2001). This implies that a neighbourhood team whose professionals personify different jobs, such as social welfare worker, nurse, psychologist and income account manager, can be seen as highly functionally heterogeneous (Keller, 2001). In contrast, a neighbourhood team can be characterised as functionally homogeneous if it consists of professionals with the same organisational role, often referred to as a generalist team. Following the study of Somech (2006), who studied functional heterogeneity in primary care teams, the present study relies on job titles to determine functional heterogeneity.

When studying functional heterogeneity, researchers commonly refer to the *information/decision‐making perspective* (Shin & Zhou, 2007; Williams & O'Reilly, 1998), which is also known as the cognitive diversity paradigm (Horwitz & Horwitz, 2007) or as elaboration‐based processes (Van Knippenberg et al., 2004). Here, the basic idea is that teams with high levels of functional heterogeneity have access to a wide range of information and perspectives, resulting in intellectual stimulation, cognitive processing and optimal use of information (Shin & Zhou, 2007). As such, functional heterogeneity is expected to enhance the process of exchanging information and perspectives among professionals in a team, the individual processing on this information, the feeding back of the results of this processing to the team and, finally, discussing and integrating the implications to improve the functioning of the team (Drach‐Zahavy & Somech, 2002; Joshi & Roh, 2009; Van Knippenberg et al., 2004). The information/decision‐making perspective thus links functional heterogeneity to the process of information elaboration, leading to our first hypothesis:

**H1:** Functional heterogeneity is positively related to information elaboration within a team.

Moreover, the information/decision‐making perspective argues that functional heterogeneity enhances access to a broader set of external networks (Ancona & Caldwell, 1992b). This means that the greater the functional heterogeneity, “the more team members communicate outside the team's boundaries” (Ancona & Caldwell, 1992b, p. 321). The second hypothesis is therefore:

**H2:** Functional heterogeneity is positively related to boundary management within a team.

So far, we have hypothesised that functional heterogeneity relates positively to both information elaboration and boundary management (Dijkhoff, 2014). The literature, however, suggests that the relationship between functional heterogeneity and cohesion is more complex (Ehrhardt, Miller, Freeman, & Hom, 2014; Tekleab, Karaca, Quigley, & Tsang, 2016). This complexity can be explained using *the social‐categorisation perspective*, which describes how people are naturally resistant to uniting with someone who they perceive as different from themselves (Chatman & Flynn, 2001; Williams & O'Reilly, 1998). In a team context, this implies that people are likely to react negatively to others with different organisational roles, thereby triggering intergroup biases (Van Dick et al., 2008; Van Knippenberg et al., 2004). These intergroup biases are associated with lower coordination capabilities and social integration (Guillaume, Dawson, Otaye‐Ebede, Woods, & West, 2017). In view of this, it could thus be argued that functional heterogeneity undermines team cohesion. Adopting the social‐categorisation perspective, the third hypothesis is therefore:

**H3:** Functional heterogeneity is negatively related to team cohesion within a team.

### 2.4 Team autonomy

It is argued that, in a team context, decisions are of better quality when they are made collectively rather than individually (Alper, Tjosvold, & Law, 1998; Baker, Day, & Salas, 2006; Johnson, 2017). Alper et al. (1998) also found that team members work more efficiently when they are collectively responsible for the decision‐making than when one authorised member is responsible for the decision‐making. As such, the perceived wisdom is that team autonomy, which entails shared decision‐making and diffused responsibilities, benefits team performance (Uhl‐Bien & Grean, 1998). In the health and social care context, team autonomy is seen as high when team members collectively manage their resources to meet the specific needs of the population they serve (Øvretveit, 1997). In contrast, when a single authorised person (e.g. supervisor) or institution (e.g. municipality) is responsible for, and held accountable for, the management of the collective resources, the team's autonomy is seen as low (Øvretveit, 1997).

The sociotechnical perspective (Clegg, 2000) explains how team autonomy specifically enhances the possibilities for members to apply knowledge and skills (Cordery et al., 2010). Consequently, members of teams with high levels of team autonomy will more strongly believe in the practical relevance of knowledge sharing (Cordery et al., 2010; Srivastava, Bartol, & Locke, 2006). Based on this logic, it has been suggested that team autonomy motivates team members to “search for solutions both within and outside the team and [for] greater collaboration [in an] attempt to help one other through knowledge sharing” (Srivastava et al., 2006, p. 1241). The search for knowledge (and its sharing) and collaboration embodies the process of information elaboration, and therefore our fourth hypothesis is:

**H4:** Team autonomy is positively related to information elaboration within a team.

Batt (1999) found that members of autonomous teams increasingly engage in external coordination and information gathering outside the boundaries of the team. Based on these findings, Batt (1999) suggested that members of autonomous teams hold each other mutually accountable for the maintenance of the team's boundaries and the communication with the team's stakeholders. Following this line of reasoning, our fifth hypothesis is:

**H5:** Team autonomy is positively related to boundary management within a team.

Team autonomy has also been discussed in the literature on trust. In this stream of literature, team autonomy is approached as being the expression of trust signalled by a third party with whom team members share a bond (such as their supervisor or the local governance) (Ferrin, Dirks, & Shah, 2006; Lau & Liden, 2008). Team autonomy therefore strengthens mutual confidence in the capabilities and priorities of team members (Ehrhardt et al., 2014). When team members experience their team as being capable of organising team processes and outcomes, they are likely to actually utilise the opportunity to make decisions collectively. This collective decision‐making subsequently signals to the individual team member that their input is valued by the other team members, strengthening mutual trust (Hoegl & Parboteeah, 2006; Srivastava et al., 2006). Team autonomy will thus result in increased mutual trust and unity through collective decision‐making (Hoegl & Parboteeah, 2006). As unity and trust both characterise team cohesion we formulate our final hypothesis as follows:

**H6:** Team autonomy is positively related to team cohesion within a team.

**Figure 1:**
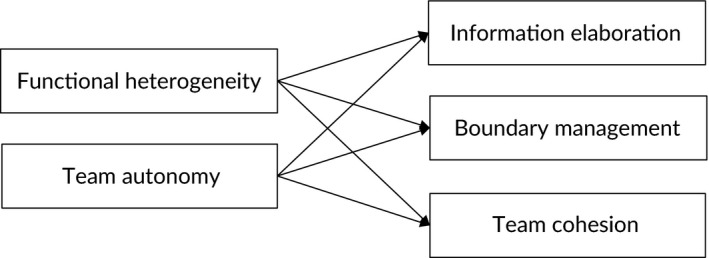
Conceptual model

Based on the literature, we have thus formulated six hypotheses that are represented in the conceptual model shown in Figure 1. Most of these hypotheses are in line with the assumptions underlying the policy programme implemented by the Dutch government. The notable exception is our third hypothesis concerning functional heterogeneity and team cohesion that would appear to run counter to the programme's assumptions. In the next step, we empirically test these six hypotheses.

## 3 METHOD

### 3.1 Sampling

Starting in May 2015, an online survey was conducted in 13 Dutch municipalities, including the four largest municipalities of the Netherlands (by the number of inhabitants). The data collection process lasted until January 2017. The networks of the researchers and convenience sampling were used to approach the 13 municipalities and their 181 neighbourhood teams. Given the organisational differences between the municipalities, the survey was adapted to the terminology of each municipality; for example “supervisor” was changed to “coordinator”, “team leader” or “coach”. In the invitation e‐mail, all respondents were informed about the purpose of the study and guaranteed anonymity. At least two reminders were sent to the professionals to improve the response rate.

### 3.2 Measures

This section describes the measurement of the variables. All the items used are listed in the Supporting Information.

*Functional heterogeneity* was calculated using Blau's (1960) index of heterogeneity, 1 − ∑ (*P*_*i*_)^2^, where P_*i*_ is the proportion of team members in the *i* th category (e.g. Wiersema & Bantel, 1992). In our study, this *i* th category represents job titles. The job titles were obtained from the municipalities’ administrations. If job titles seemed similar, we evaluated the job descriptions by studying corresponding vacancy adverts and, if the different job titles represented the same job, they were assigned to the same category. If not, new categories were added. Following this process, we identified a total of 39 job titles in our sample. If less than 85% of the team members’ job roles were available in the administrative data, the score of functional heterogeneity was labelled as missing (*n *=* *6). All in all, by evaluating administrative data, we were able to develop an objective measure of functional heterogeneity in a similar way to Somech (2006). The heterogeneity index can range between 0 and 1 (Blau, 1960) and the minimum and maximum values in our sample were 0 and 0.98 with an average heterogeneity of 0.52.

*Information elaboration* was measured by means of five items in the survey based on the information elaboration scale of Van Dick et al. (2008). An example item being “In my neighbourhood team, we discuss the content of our work a lot”. The responses were given on a 5‐point Likert scale ranging from “1” fully disagree to “5” fully agree. This scale was also used for the other measures. The scale reliability was good based on the calculated Cronbach's alpha of 0.904.

*Boundary management* was evaluated by means of five items in the survey inspired by Ancona and Caldwell's (1992a) measures for boundary activity. An example item is “My team members convince relevant stakeholders in the neighbourhood (like the police, general practitioners, housing corporations and welfare authorities) that the team's activities are important”. The Cronbach's alpha was 0.933.

*Team cohesion* was also assessed by means of five items in the survey, this time inspired by the measurement scale of Carless and De Paola (2000). An example item is “In my neighbourhood team we are united in trying to reach our goals for team performance”. The Cronbach's alpha for this scale was 0.916.

*Team autonomy* was similarly measured by five items, based on Campion, Medsker, and Higgs (1993) measurement scale for self‐management. An example item being “In my neighbourhood team, we allocate the tasks ourselves”. The Cronbach's alpha was 0.835.

*Control variables* included in the study were team size and team tenure. Team size is seen as influencing team processes in that a large team leads to coordination and control issues (Smith, Smith, Olian, & Sims, 1994) and less participation (Poulton & West, 1999). Team sizes were obtained from the municipalities’ administrations and were between 4 and 43. The logarithm of team size was included in the model as a control variable.

Team tenure was established as the number of months between January 2015 (the introduction of the policy programme) and when a team was included in the study. Team tenure influences team processes as “team composition–outcome relationships are likely to be variable over time and need to be considered” (Mathieu et al., 2014, p. 146). The teams in the present study had tenures between 17 and 27 months, and the logarithm of tenure has been included in the model as a control variable.

### 3.3 Data analyses

In order to test the hypotheses, the individual scores needed to be aggregated to the team level. To evaluate whether data aggregation is justified, the *R*_wg_ and the intraclass correlations (ICC1 and ICC2) were evaluated. To calculate the ICCs, we *estimated* an ‘average’ team size (to take account of the relatively wide range of team sizes—between 4 and 43) (Bliese & Halverson, 1998, p. 168) using the following formula:

$$\begin{array}{cc} \mathrm{Ng} & =\left(1/\text{(Number of teams}-1\right))\times \left(\sum \text{Team sizes}-\left(\sum {\text{Teamsizes}}^{2}/\sum \text{Team sizes}\right)\right)\\ & =(1/(170-1))\times (2584-(47330/2584))=15.18. \end{array}$$

In the results shown in Table 1, all the R_wg_ values are above 0.7 and the ICC1 values fall within the typical range of 0.05 to 0.20 with significant F‐values. As such, aggregation is justified (Bliese, 2000).

**Table 1:** Intraclass correlations (*n *=* *1335)

	*R*_wg_	ICC1^a^	ICC2^b^	*F* c
Information elaboration	0.79	0.05	0.43	1.76^d^
Team Cohesion	0.81	0.09	0.60	2.50^d^
Boundary management	0.79	0.08	0.58	2.38^d^
Team autonomy	0.81	0.08	0.57	2.31^d^

*Note*. ICC = intraclass correlation; MSB =* *mean square between teams; MSW = mean square within teams; k = estimated team size.

a ICC1 = (MSB‐MSW)/(MSB + (k‐1) x MSW)).

b ICC2 = (MSB‐MSW)/MSB.

c F = MSB/MSW; *df*(within) = 1165; *df*(between) = 169.

d *p *< 0.01.

The hypotheses were tested using structural equations modelling (SEM). Following the recommendations of Anderson and Gerbing (1988), we first examined the measurement model to ensure that our various constructs were distinctive (corresponding to a CFA). The CFA and SEM with robust maximum‐likelihood estimation and a Satorra–Bentler scaled difference test were run in Rstudio^®^ version 1.0.136 using the Lavaan (Rosseel, 2014) and semPlot (Epskamp, 2015) packages. The global fit between the model and the observed data was evaluated using three absolute fit indices: the chi‐square “goodness‐of‐fit” test (*χ*²), the standardised mean square residual (SRMR) and the root‐mean‐squared error of approximation (RMSEA) (Brown, 2015). A nonsignificant χ² value with a *χ*²/*df* value below 2, an SRMR value equal or below 0.08 and an RMSEA equal or below 0.08 indicates an acceptable model fit (Anderson & Gerbing, 1988; Brown, 2015; Browne & Cudeck, 1993; Hu & Bentler, 1999). Additionally, two relative fit indices were evaluated: the comparative fit index (CFI) (Bentler, 1990) and the Tucker–Lewis index (TLI) (Tucker & Lewis, 1973). The threshold values for a good model fit are CFI and TLI values greater than 0.9 (Hu & Bentler, 1999). In terms of local model fit, model misspecification can be identified by evaluating the factor loadings, modification indexes (MI) and expected parameter changes (EPC) (Brown, 2015). A misspecification is more specifically indicated by the standardised factor loadings being nonsignificant and/or below 0.4, MI values being 3.84 or greater and EPC values above 0.2 (Brown, 2015). In such instances, model improvements were made.

### 3.4 Ethics approval and consent to participate

This study is based on one single anonymous survey, which was free from radical, incriminating, or intimate questions. Completion was possible within a reasonable time period of approximately twenty minutes and participation in the survey was voluntary. All participants (i.e. professionals) were considered to be competent. Ethical approval was therefore not required under Dutch law on medical research (Medical Research Involving Human Subjects Act, http://www.ccmo.nl).

The obtained responses were stored separately from the personal details, and it was impossible to link individual responses with participant's identities. Complete confidentiality and anonymity was guaranteed to the participant. The data processing was therefore accordance the Dutch Personal Data Protection Act (http://www.privacy.nl/uploads/guide_for_controller_ministry_justice.pdf).

## 4 RESULTS

In total 1,400 of the 2,584 professionals working in the 181 neighbourhood teams included in our study completed the online survey (a 54% response rate). The minimum of responding team members for inclusion in the study was set on 30%. Eleven teams did not reach this threshold, resulting in the inclusion of 1,335 professionals working in 170 teams. The respondents’ characteristics are reported in Table S1 in the Supporting Information. Compared to the overall population in the social domain, our sample includes more women than in the population of social workers (85% against 76%) and specialists (76%) (CBS StatLine, 2018). Table 2 presents the means, standard deviations and correlations of the team averages. Table 2 shows that, in line with the literature, information elaboration, boundary management and cohesion correlated positively with team autonomy. However, in contrast to the literature, functional heterogeneity correlated negatively with information elaboration. In line with literature suggestions, team size correlated negatively with team autonomy, information elaboration, and cohesion. Finally, team tenure correlated negatively with functional heterogeneity, boundary management and team size. A possible explanation for these negative correlations is that in the beginning phase of the data collection period the teams were functionally more heterogeneous. Given that almost all the bivariate correlations are below 0.7 (Table 2) and the corresponding Variation Inflation Factors (VIF) (Table S2 in the Supporting Information) are below 10 it seems that the data are not subject to multicollinearity (Field, 2013).

**Table 2:** Means, standard deviations and correlations

		*N*	Mean	*SD*	1	2	3	4	5	6
1	Functional heterogeneity	164	0.52	0.34						
2	Team autonomy	170	3.74	0.35	−0.20**					
3	Information elaboration	170	3.77	0.33	−0.26**	0.66**				
4	Boundary management	170	3.64	0.37	−0.06	0.38**	0.58**			
5	Team Cohesion	170	4.03	0.37	0.02	0.68**	0.71**	0.60**		
6	Team size^a^	170	1.14	0.18	0.13	−0.23**	−0.34**	−0.10	−0.23**	
7	Team tenure^a^	170	1.30	0.08	−0.50**	0.18*	0.05	−0.28**	−0.09	−0.21**

a Logarithm.

*p <* *0.05; **p <* *0.01 = significant *p* values.

On evaluation, the initial measurement model yielded an unsatisfactory model fit: χ² (164) = 367, *p* < 0.01, SRMR = 0.052, RMSEA = 0.092 (90% CI 0.079–0.104, Cfit < 0.05), TLI = 0.899, CFI = 0.912 (Table 3). To improve the model fit, the modification indices (MI's) and expected parameter change (EPC) values were evaluated, and after theoretical reasoning (Arbuckle, 2012, p.110) three error term correlations were added. First, improved model fit (MI = 97.11, EPC = 0.05) was suggested for correlating the error terms between the fourth and the fifth item of cohesion, which were the only items measuring attitudes instead of behaviours. Next, improved model fit (MI = 16.32, EPC = 0.02) was suggested for correlating the error terms between the third and the fourth item of information elaboration (MI = 17.31, EPC = 0.02). These error term correlations were added because only these two items started with “In my neighbourhood team”. Last, an error term correlation was added between the second and fifth item of information elaboration (MI = 28.07, EPC = 0.02) because both items include a description of team members who say something “new”.

**Table 3:** Goodness‐of‐fit test results for each model

	*χ*² (*df*)	*χ*²/*df*	RMSEA	SRMR	CFI	TLI	AIC	BIC	*χ*²diff
Measurement model^a^									
Baseline model	2132 (190)	11.22							
Theoretical model	367(164)	2.24	0.092	0.052	0.912	0.899	1253	1460	
Revised model	252 (161)	1.57	0.062	0.046	0.961	0.954	1124	1341	83(3)^c^
Structural model^b^									
Revised model	403 (212)	1.90	0.078	0.077	0.923	0.909	682	924	
Fit criteria good fit		≤2.00	<0.08	<0.08	>0.9	>0.9	Smaller values indicate a better model		

a *N *=* *170.

b Listwise *N *=* *164.

c *p *<* *0.001.

The revised measurement model provided an adequate fit: *χ*² (161) = 252, *p *< 0.01, SRMR = 0.046, RMSEA = 0.062 (90% CI 0.047–0.076, Cfit < 0.05), TLI = 0.954, CFI = 0.961 (Table 3). The scaled difference in *χ*² values was tested (Satorra & Bentler, 1994) and this showed that the fit of the measurement model had significantly improved after the modifications (*χ*²diff (3)= 83.37,  *p*< 0.001). To build our structural model we then added the regression coefficients, the independent variable functional heterogeneity and the team size and team tenure control variables. The default for missing values in the Lavaan (Rosseel, 2014) and semPlot (Epskamp, 2015) packages is listwise deletion, which means that only the complete data are used. On evaluation, this structural model was just acceptable: *χ*² (212) = 403, *p* < 0.01, SRMR = 0.077, RMSEA = 0.077 (90% CI 0.066–0.089, Cfit <0.05), TLI = 0.909, CFI = 0.923 (Table 3).

The regression coefficients in the structural model indicate that functional heterogeneity is negatively related to information elaboration (*β* = −0.27, *p* < 0.001) and to boundary management (*β* = −0.22, *p* < 0.05), and unrelated to cohesion (*β *= 0.05, *p* > 0.05). The regression coefficients further indicate that team autonomy is positively related to information elaboration (*β* = 0.77, *p* < 0.001), to boundary management (*β *= 0.47, p < 0.001) and to cohesion (*β *= 0.87, *p* < 0.001). An overview of the estimates is provided in Table 4.

**Table 4:** Regression estimates (*N *=* *164)

	Information elaboration	Boundary management	Team Cohesion
Functional heterogeneity	−0.27***	−0.22*	0.05
Team autonomy	0.77***	0.47***	0.87***

*Note*. Control variables included in the study are team size and team tenure.

*p <* *0.05; ***p <* *0.001 =  significant *p* values.

## 5 DISCUSSION AND CONCLUSION

The central research question of our study was: To what extent do functional heterogeneity and team autonomy influence informational elaboration, boundary management and team cohesion in Dutch neighbourhood teams? In answering this research question, we now discuss the study's findings (in the order of the hypotheses). We then conclude the article by relating the findings to the literature, discussing the limitations of our study and considering the present study's implications for the literature and the policy programme.

First, in contrast to our initial hypothesis (H1), the results show a negative relationship between functional heterogeneity and information elaboration. Related to this finding, we saw that the respondents frequently took advantage of the opportunity to give an open answer and indicated that they lacked information on their colleagues’ knowledge and expertise. This “knowing who knows what” is part of a team's *transactive memory* (Oshri, Fenema, & Kotlarsky, 2008; Van Knippenberg et al., 2004). It seems likely that transactive memory is, at least initially, higher in functionally homogenous teams than in heterogeneous teams given that professionals from similar disciplines will have shared information from previous education, training or work experiences (Ehrhardt et al., 2014). Continuing this reasoning, transactive memory could thus mediate the effect of functional heterogeneity on information elaboration. Further research is however needed to test the validity of this mediated relationship.

Interestingly, in contrast to our second hypothesis (H2), the results also show a negative relationship between functional heterogeneity and boundary management. This opposes the idea that professionals communicate more with those outside the team's boundaries when their team includes a greater range of job roles (Ancona & Caldwell, 1992b; Keller, 2001). Moreover, this negative relationship between functional heterogeneity and boundary management indicates that a greater variety of job roles hinders teams to manage their external relationships. A possible explanation could be that team members separate themselves based on their job roles (Chatman & Flynn, 2001; Williams & O'Reilly, 1998), which leads to subgroups within functionally heterogeneous teams. These subgroups subsequently harm the single team functioning, with the risk of becoming a loosely coupled group rather than a team. As management of external relationships is an important characteristic that distinguishes teams from groups (Cohen & Bailey, 1997, p. 241), functional heterogeneity thus possibly hinders boundary management through disintegration of the team. Altogether, we invite future researchers to examine the mediating role of subgroups within the relationship between functional heterogeneity and boundary management.

Next, we failed to find a relationship between functional heterogeneity and team cohesion (H3). This suggests that there may be additional team characteristics or processes that play a role in the relationship between functional heterogeneity and team cohesion. We would therefore encourage future researchers to examine this relationship by including theory‐based moderators and/or mediators.

Finally, conforming our final group of hypotheses (H4, H5 and H6), team autonomy relates positively with information elaboration, boundary management and team cohesion. This suggests that team autonomy is a powerful team characteristic with which to improve team processes and emergent states in neighbourhood teams. Moreover, the effects of team autonomy were stronger than those of functional heterogeneity (Table 4).

We thus answer our research question by concluding that the strongest positive influence on information elaboration, boundary management and team cohesion comes from team autonomy, on top of which an additional negative influence on information elaboration and boundary management come from functional heterogeneity.

### 5.1 Limitations

The present study has several limitations. First, this study relied on the analysis of mainly cross‐sectional self‐reported data. This means that the study's findings could be subject to common method bias. Following the procedure of Podsakoff, MacKenzie, Lee, and Podsakoff (2003), we controlled for common method variance through an ex ante procedural remedy (i.e. applying functional heterogeneity from a different source) and ex post statistical controls (i.e. testing whether a model with unmeasured common method variance fits significantly better) (Podsakoff et al., 2003). The scaled difference in χ² (Satorra & Bentler, 1994) between the revised measurement model and the common method variance model was insignificant (Table S3 in the Supporting Information), indicating that the relationships in our model are very unlikely to be inflated by common method bias (Conway & Lance, 2010; George & Pandey, 2017). Furthermore, the cross‐sectional character of our data limits the possibilities to make causal inferences. Nevertheless, cross‐sectional studies are viewed as being sufficiently powerful to identify and verify relationships that have not been previously tested (Spector, 2006).

Second, our results revealed relatively low ICC values which could mean that our findings are attenuated (Bliese, 2000). These low values suggest that there was only a limited consensus in the responses of the professionals within individual neighbourhood teams. Thus, although we had theoretical arguments to aggregate our data, future research could further investigate the causes of the individual variability within neighbourhood teams. This is in line with the argument of Van Knippenberg and Mell (2016) who observe that, although team processes are typically measured as a shared perception, studying the differences in perceptions could provide more relevant information than studying the mean perception of team processes.

Third, our results suggest a negative relationship between functional heterogeneity and information elaboration. Given that this was contrary to our theoretical expectations, we argued that transactional memory might be an important mediator in this relationship. However, we lack quantitative measures of transactional memory to test this suggestion. We therefore encourage future researchers to develop a short one‐dimensional measurement scale for transactional memory (such as a shortened version of the fifteen‐item three‐dimensional scale of Lewis (2003)) to examine the mediating effect of transactive memory in neighbourhood teams.

### 5.2 Implications

These limitations notwithstanding, our study has theoretical and practical implications. The present study adds knowledge to the current academic debate in at least two ways. The first contribution is to the overall team literature by answering calls for research into team processes and emergent states. Based on our results, the present article offers a theoretical foundation for new propositions on how team autonomy in particular can improve team outcomes through information elaboration, boundary management and cohesion. As such, our first contribution is in offering preliminary insights into how team autonomy improves team processes and emergent states in the context of neighbourhood teams.

The second contribution of this study is to the team diversity literature by testing the effect of functional heterogeneity on information elaboration. In this stream of literature, it is frequently theorised that functional heterogeneity achieves beneficial team outcomes through the process of information elaboration (Van Knippenberg et al., 2004; Van Knippenberg & Mell, 2016). However, our study illustrates that functional heterogeneity can also hinder information elaboration. This supports the idea that team members first need to learn how to translate their differences into beneficial outcomes (Van Knippenberg et al., 2004). Future researchers are therefore encouraged to test additional moderating mechanisms (Van Knippenberg & Mell, 2016). Our study thus contributes to the current theoretical debate on the relationship between team diversity and outcomes by questioning the positive relationship between functional heterogeneity and information elaboration.

Ultimately, our study has at least one practical implication. In the introduction we have reconstructed the assumptions underlying the policy programme. Examination of these assumptions revealed both strengths and limitations of the programme. On the one hand, our findings cast some doubts over the effectiveness of functional heterogeneity in neighbourhood teams. On the other hand, our findings suggest that team autonomy is a powerful intervention that can increase information elaboration, boundary management and cohesion in neighbourhood teams. Therefore, given that information elaboration, boundary management and cohesion will lead to better team performance (Kuipers & Groeneveld, 2014), policy makers or supervisors who wish to improve or maintain high performance in a neighbourhood team should organise, encourage and support team autonomy.
