# Supplementary material for: Towards sustainable local welfare systems: The effects of functional heterogeneity and team autonomy on team processes in Dutch neighbourhood teams
Source: Health Soc Care Community. 2018 Jul 26;27(1):82–92. doi: 10.1111/hsc.12604 (PMC7379639; doi:10.1111/hsc.12604)
Supplement: Supplementary file 1 [file HSC-27-82-s001.pdf]

Supporting Information

# Towards sustainable local welfare systems: The effects of functional heterogeneity and team autonomy on team processes in Dutch neighbourhood teams

*Van Zijl, A.L., Vermeeren, B., Koster, F., & Steijn, B.*

*Erasmus University Rotterdam, Department of Public Administration and Sociology, Erasmus University Rotterdam, P.O. Box 1738, 3000 DR Rotterdam, Netherlands.*

*Email: vanzijl@essb.eur.nl*

**Content**

Survey items

Table S1

Table S2

Table S3

Supporting Information

Survey items

*Information elaboration*

1. My team members exchange a lot of information about the task.
2. My team members often say things that lead me to learn something new about the job.
3. In my neighbourhood team, we discuss the content of our work a lot.
4. In my neighbourhood team, we often talk about our ideas about the task.
5. My team members often say things that lead me to new ideas.

*Boundary management*

1. My team members make contact with relevant stakeholders in the neighbourhood (like the police, general practitioners, housing corporations and welfare authorities).
2. My team members inform relevant stakeholders in the neighbourhood (like the police, general practitioners, housing corporations and welfare authorities) about the working methods of our team.
3. My team members proactively ask relevant stakeholders in the neighbourhood (like the police, general practitioners, housing corporations and welfare authorities) for advice and support.
4. My team members convince relevant stakeholders in the neighbourhood (like the police, general practitioners, housing corporations and welfare authorities) that the team's activities are important.
5. My team members keep relevant stakeholders in the neighbourhood (for example the police, the general practitioner, housing corporations and welfare authorities) informed of our team's activities.

Supporting Information

*Team cohesion*

1. In my neighbourhood team, we are united in trying to reach our goals for team performance.
2. In my neighbourhood team, we take our responsibilities for setbacks or poor team performances.
3. In my neighbourhood team, we help each other to perform the tasks.
4. In my neighbourhood team, we get along well together.
5. In my neighbourhood team, we trust each other.

*Team autonomy*

1. In my neighbourhood team, we are involved in work-related decision-making.
2. In my neighbourhood team, we comment on work-related issues.
3. My neighbourhood team contributes to the policy development on working in my neighbourhood team.
4. In my neighbourhood team, the work-related decisions are made collectively.
5. In my neighbourhood team, we allocate the tasks ourselves.

Supporting Information

Table S1

*Descriptive statistics and participants characteristics*

| | Variable | Mean | S.D. | Minimum | Maximum | N |
|---|---|---|---|---|---|---|
| 1 | Functional heterogeneity | .52 | .34 | 0 | .98 | 164 |
| 2 | Team autonomy | 3.74 | .35 | 2.78 | 4.47 | 170 |
| 3 | Information elaboration | 3.77 | .33 | 2.71 | 4.68 | 170 |
| 4 | Boundary management | 3.64 | .37 | 2.76 | 4.46 | 170 |
| 5 | Cohesion | 4.03 | .37 | 3.06 | 4.80 | 170 |
| 6 | Logarithm team size | 1.14 | .18 | .6 | 1.63 | 170 |
| 7 | Team size | 15.20 | 6.89 | 4 | 43 | 170 |
| 8 | Logarithm team tenure | 1.30 | .078 | 1.23 | 1.43 | 170 |
| 9 | Team tenure in months | 20.24 | 3.80 | 17 | 27 | 170 |
| 10 | Female (dummy: yes) | .85 | | 0 | 1 | 1335 |
| 11 | Age in years | 41.41 | 9.79 | 20 | 65 | 1330 |
| 12 | Professional tenure in years | 14.41 | 9.79 | 0 | 45 | 1321 |
| 13 | Education level | 5.15 | .54 | 1 | 7 | 1335 |
| 14 | Working hours per week | 30.53 | 4.92 | 4 | 40 | 1331 |

Supporting Information

Table S2

*Collinearity Statistics*

| | Variable | tolerance | VIF |
|---|---|---|---|
| 1 | Functional heterogeneity | 0.74 | 1.35 |
| 2 | Team autonomy | 0.92 | 1.09 |
| 3 | Team size | 0.92 | 1.08 |
| 4 | Team tenure | 0.73 | 1.36 |

*Note.* Tolerance values below .1 Variance Inflation Factor (VIF) values above 10 are indicative of serious multicollinearity problems

Supporting Information

Table S3

*Goodness-of-fit test results for each model including the common method model*

| | $\chi^2$ (df) | $\chi^2$ /df | RMSEA | SRMR | CFI | TLI | AIC | BIC | $\chi^2_{diff}$ |
|---|---|---|---|---|---|---|---|---|---|
| Measurement model | | | | | | | | | |
| Revised model | 252 (161) | 1.57 | .062 | .046 | .961 | .954 | 1124 | 1341 | |
| Common method factor | 247 (159) | 1.55 | .061 | .055 | .963 | .955 | 1114 | 1340 | 4 (2) |
| Criteria for good fit | | ≤2.00 | <0.08 | <0.08 | >0.9 | >0.9 | Smaller values indicate a better model | | |

*N*=170

Supporting Information
